# Comparison of Doppler ultrasound of the milk vein and the Fick principle to estimate mammary plasma flow on the day of calving and at 7 days in milk in multiparous Holstein cows*

**DOI:** 10.3168/jdsc.2026-1036

**Published:** 2026-06-11

**Authors:** Amanda J. Fischer-Tlustos, Kayleigh Klein, Julia Petrou, Cadie McQuaig, Dave J. Seymour, Eduardo Ribeiro, Juliana Mergh Leao, Colby A. Redifer, Allison M. Meyer, John P. Cant, Michael A. Steele

**Affiliations:** 1 Department of Animal Biosciences, Animal Science and Nutrition, University of Guelph, Guelph, ON, Canada N1G 1Y2; 2 Trouw Nutrition Research and Development, 5830 AE Boxmeer, the Netherlands; 3 The Saskatoon Colostrum Company Ltd., Saskatoon, SK, Canada, S7K 6A2; 4 Division of Animal Sciences, University of Missouri, Columbia, MO 65211

## Abstract

The aim of this study was to describe the utility of Doppler ultrasonography of the milk vein (MV) to assess mammary plasma flow (MPF) and determine whether a relationship exists between MPF assessed via Doppler ultrasound of the MV (MPF_MV_) and the Fick principle (MPF_Fick_) on the day of calving and at 7 DIM. A total of 11 parity-2 (P2) and 10 parity-3 (P3) Holstein cows were enrolled in the study immediately after calving. The MPF_Fick_ was estimated using the mammary arteriovenous difference of Phe and Tyr as internal markers at 2, 4, 6, and 8 h after colostrum collection and the morning milking at 7 ± 1 DIM. Milk was collected at milking 2 and the evening milking at 7 ± 1 DIM for determination of milk protein output. The MPF_MV_ was also estimated by Doppler ultrasound of the left and right MV at 2 and 8 h after colostrum collection and the morning milking at 7 ± 1 DIM. Both MPF_MV_ and MPF_Fick_ increased by 15.8% and 19.5% from calving to 7 DIM, respectively. No differences were detected between parity in MPF_Fick_; however, P3 cows had greater MPF_MV_ than P2 cows, as well as greater MV diameter. No relationship existed between MPF_MV_ and MPF_Fick_ during the calving and fresh periods, which may have been due to the violation of multiple assumptions of the Fick principle as a result of the fluctuating rate of milk secretion, tissue metabolism, and blood flow associated with the onset of lactation. Although Doppler ultrasound of the MV may be a time-efficient, noninvasive technique to estimate fresh-period MPF, further work is needed to validate its accuracy with techniques estimating MPF directly in the pudendal artery, such as the dye dilution technique. Such knowledge would allow for the widespread utilization of this noninvasive technique to uncover knowledge gaps pertaining to the relationship between fresh-period mammary metabolism and milk production.

## Graphical Abstract

**Figure:**
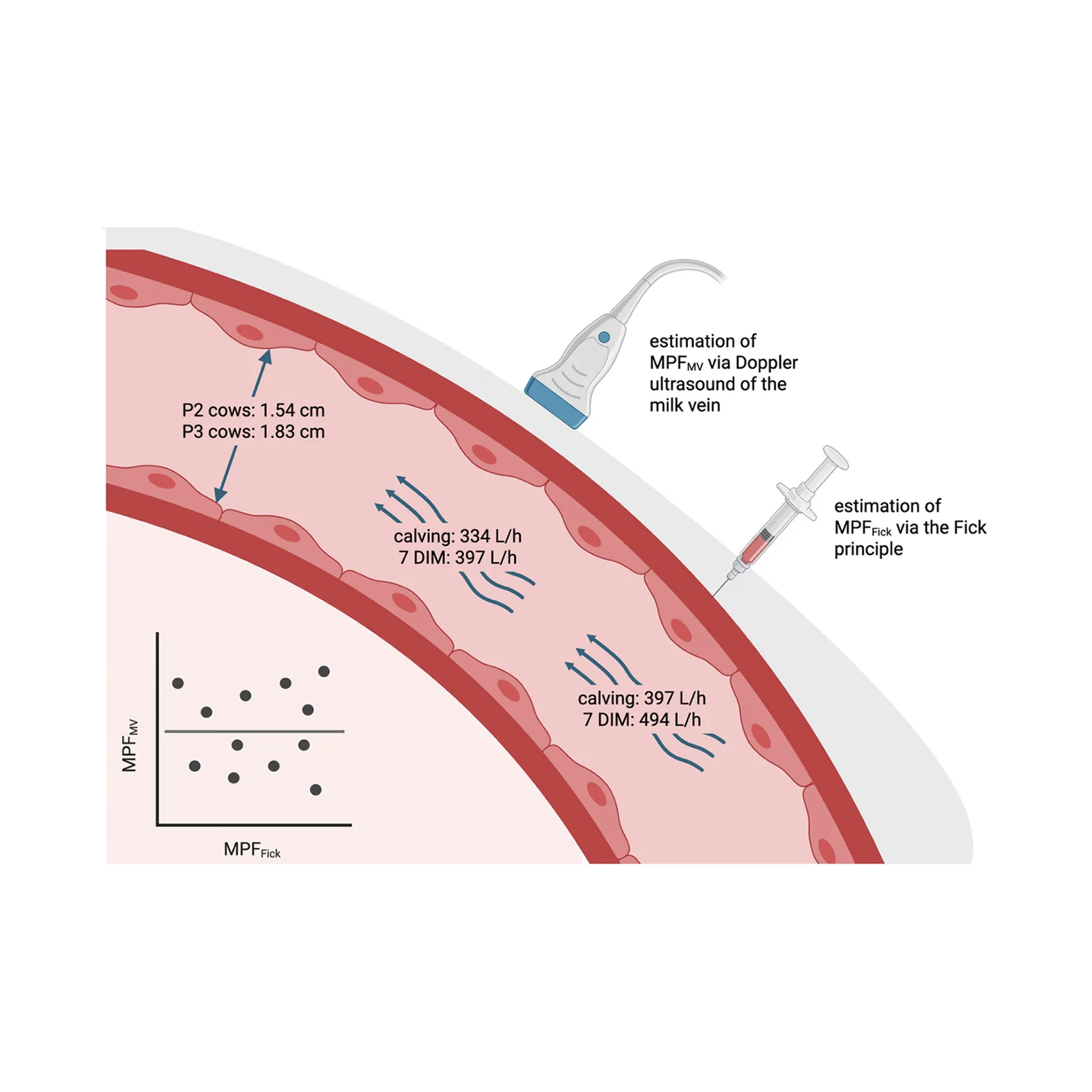

The concept of functional hyperemia states that as the metabolic activity of a tissue increases, so too does its blood flow rate (Johnson, 1978). Thus, mammary functional hyperemia infers that as the metabolic activity and ATP requirement of the gland increase, mammary plasma flow (**MPF**) will increase to supply energetic substrates to meet this demand (Cant et al., 2018). As such, the level of mammary metabolic activity is mirrored in the local blood flow rate. The onset of lactation is characterized by a rapid increase in metabolic activity of the mammary glands related to milk synthesis (Baldwin, 1966). The study of MPF at this time can therefore generate insights into how prepartum or early-lactation interventions influence milk production and the establishment of a successful lactation.

Ultrasonic flow probes, thermocouples, and dye dilution techniques have been previously used to assess MPF; however, local or general anesthesia is required to allow access to the pudendal arteries supplying the mammary glands. The Fick principle is a commonly used alternative to assess MPF in the mammary arterial supply, as it requires only the collection of frequent pairs of mammary arteriovenous difference (**AVD**) samples and a measure of milk protein output. The accuracy of the Fick principle has been validated against more direct estimates of MPF, such as pudendal artery electromagnetic and ultrasonic flow probes, in mid- to late lactation (Davis et al., 1988; Thivierge et al., 2002). Unfortunately, this technique still subjects the animal to stress due to frequent venipuncture of the coccygeal and caudal superficial epigastric veins (milk vein; **MV**), which is undesirable when the cow is simultaneously undergoing physiological, immunological, and metabolic stress associated with the onset of lactation. Transrectal Doppler ultrasonic measurement of the pudendal artery is a possible alternative; however, this technique is time-consuming, still invasive, and undesirable in the days following calving due to welfare concerns. Noninvasive MV blood flow rates were determined via Doppler ultrasound as early as 1989 by Christensen et al. (1989) in lactating goats, with later work using this technique to diagnose clinical abnormalities in Swiss Braunvieh and Simmental cattle daily from 9 d prior to calving, within 3 h of calving, and up to 10 DIM (Braun et al., 2013) and to assess the relationship between hepatic and mammary blood flow (Themistokleous et al., 2023a) and between udder health and productivity and mammary blood flow (Themistokleous et al., 2023b) in second- and third-lactation Holstein cattle throughout late lactation, the dry period, the day of calving, and early lactation. However, no attempts have been made to validate Doppler ultrasound of the MV as an estimate of MPF throughout the immediate postcalving and early-lactation periods through characterization of its relationship with more common methods of assessing MPF, such as the Fick principle.

It was hypothesized that a positive, linear relationship would exist between MPF as estimated by Doppler ultrasound of the MV (**MPF_MV_**) and the Fick principle (**MPF_Fick_**) during the immediate postcalving and fresh periods. Thus, the objectives of this study were to (1) describe the utility of Doppler ultrasonography to assess fresh-period MPF and (2) determine whether a relationship exists between MPF_MV_ and MPF_Fick_ during the fresh period.

All experimental procedures were conducted in accordance with the Canadian Council of Animal Care (1993) and were preapproved by the University of Guelph Animal Care and Use Committee for Livestock (AUP #4475). A total of 11 parity-2 (**P2**) and 10 parity-3 (**P3**) cows with a milk SCC <200,000 cells/mL for >1 mo prior to dry-off and no history of mastitis in any previous lactation were enrolled in the study. The average previous-lactation peak milk yield (**MY**), total milk production, and 305-d mature-equivalent (**305ME**) milk yield were 36.8 ± 3.62 and 46.3 ± 3.57 kg, 10,354 ± 1,172 and 13,464 ± 2,184 kg, and 9,663 ± 740 and 11,809 ± 1,098 kg for P2 and P3 cows, respectively. The selected number of animals was determined from the SD of MV ultrasound blood flow rates (SD = 72 L/min; Braun et al., 2013) to provide a power of 0.80 to detect a 50 L/h difference in MPF_MV_ from calving to 7 DIM at a level of significance of 0.05. For the entirety of the sampling period, all cows were housed in individual maternity pens and fed a standard lactating TMR composed of 1.70% wheat straw, 31.2% corn silage, 31.2% alfalfa silage, 21.6% high moisture corn and 14.2% lactating cow supplement, supplying 54.4 Mcal and 1,662 g of ME and MP per day, respectively, as described by Fischer-Tlustos et al. (2026).

To estimate MPF_Fick_, blood was collected by needle puncture from a coccygeal vessel, representative of arterial blood, and the left MV at 2, 4, 6, and 8 h after milking at 0 DIM (1.4 ± 0.76 h after calving) and after the morning milking at 7 ± 1 DIM. Serum was collected after clotting and centrifugation at 3,000 × *g* for 15 min at 4°C and frozen at −20°C until determination of Phe and Tyr by ultra-performance liquid chromatography (Bidlingmeyer et al., 1984; Waters Corporation, Milford, MA). Amino acids were derivatized using the AccQ-Tag Ultra derivatization kit (Waters Corporation), and AA peak areas from samples were compared with standard peak areas to determine concentrations using Waters Empower 2 Software (Waters Corporation). At the end of the blood sampling period, cows were milked (12.1 ± 0.49 h after colostrum collection and 11.7 ± 0.31 h after morning milking at 7 DIM), yield was recorded, and milk samples were frozen at −80°C. Milk N was determined via Dumas combustion (LECO-FP 828 analyzer; LECO Instruments, Mississauga, ON, Canada), and milk CP was calculated as N × 6.38. The MPF_Fick_ across the whole udder was estimated according to the Fick principle. The Phe and Tyr AVD were averaged across all 4 replicates per milking interval. Milk output of Phe + Tyr was estimated using the milk protein yield at milking 2 and the evening milking at 7 DIM, after subtracting IgG yield as analyzed by radial immunodiffusion (Fischer-Tlustos et al., 2026), assuming a Phe + Tyr content of 0.748 mmol/g protein and a 3.5% contribution from blood-derived proteins (Lapierre et al., 2012). As described by Cant et al. (1993), the following equation was used to calculate MPF_Fick_:

$$MPF_{Fick}\;\left(L/h\right)\;=\frac{\left[0.965\;\times \left[milk\;Phe+Tyr\;output\;\left(mmol/h\right)\right]\right]}{Phe+Tyr\;AVD\;\left(mmol/L\right)}.$$

On the same days as MPF_Fick_ estimation, an Esaote MyLab Delta Ultrasound System (Esaote, Fishers, IN) equipped with a SV3513 linear probe (8 cm in length; Esaote) was used to determine MV diameter and time-averaged mean velocity (**TAV**) of blood flow in the left and right MV. Prior to MPF_Fick_ sample collection at 0 and 7 DIM, ultrasound measurements were performed 2 and 8 h after milking. Cows were locked in headgates and acclimated to their surroundings for 2 to 5 min. Due to the substantial curvature and tortuosity of the MV immediately cranial to the udder, the first straight 20-cm segment of the MV from the cranial base of the udder was used for MPF_MV_ estimation, as described by Themistokleous et al. (2023a,b). The distance from the cranial base of the udder was measured along the MV using a flexible measuring tape, and the position was recorded (average distance = 39.1 ± 6.91 cm) and used during repeated samplings for each respective MV per cow. Although there is potential for mixing of nonmammary, subcutaneous venous blood at this position, mammary venous blood flow volume is expected to predominate (∼90% of total blood flow volume as estimated by Braun and Forster, 2012) in lactating cattle. The straight 20-cm segment was shaved, ultrasound gel was applied, and the probe was applied parallel to the MV, with care taken not to compress the vein. It is important to note that in previous work (Braun and Forster, 2012; Themistokleous et al., 2023a,b) MV diameter has been estimated using cross-sectional placement of the probe. During technique development, it was noted that cross-sectional placement of the probe resulted in a high CV (10.7%) between replicates compared to parallel placement (1.8%); thus, all measurements, including MV diameter, were conducted in parallel placement. The MV diameter was first examined in B-mode to determine MV diameter. The pulsed-wave mode was then used to determine MV TAV, as shown in Supplemental Figure S1 (see Notes). An angle of insonation of 60° was used to avoid overestimation of measurements (Arning, 2002). For each individual replicate, the sample diameter was set to 80% of the MV diameter initially measured in B-mode, as it was noted during protocol development that a diameter slightly greater than the probe's maximum sample diameter (2.4 cm) was possible. An optimal spectral tracing was achieved, and TAV was measured during a 5-s clip. The ultrasound was reverted to B-mode and the total MV diameter was measured again to calculate MV area to determine the total MV blood flow rate. The probe was removed, and the procedure was repeated a maximum of 5 times per MV, or until a minimum of 3 MPF_MV_ measurements were achieved with a CV <20% (average CV = 11.3%). The protocol was then repeated for the opposite MV, with the entirety of the procedure lasting ∼15 to 20 min. Across the 3 accepted replicates per MV per timepoint, the MV diameter and TAV were averaged. The MPF_MV_ for each replicate was calculated as follows:

$$MPF_{MV}\;\left(L/h\right)=\frac{TAV\;\left(cm/s\right)\times area\;\left(cm^{2}\right)\times 3,600}{1,000}\times 0.70,$$

where the factor of 0.70 represents a packed cell volume of 30% in dry and lactating cows (Reynolds et al., 2003).

It is important to note that for an unrelated experimental objective, 6 P2 cows and 5 P3 cows were subjected to a core mammary biopsy on alternating rear quarters at d −35, −21, and −7 relative to expected calving, 11.1 ± 5.04 min after colostrum collection (1.6 ± 0.61 h after calving), and after the evening milking at 7 DIM, as described by Daley et al. (2018). After colostrum collection, a mammary biopsy was conducted on the rear right quarter, and the subsequent blood samples for MPF_Fick_ estimation were collected only on the left MV. At 7 DIM, mammary biopsy was conducted after performing both MPF_MV_ and MPF_Fick_ measurements.

Variance, correlation, and regression analyses were conducted using SAS Studio (v. 9.4, SAS Institute Inc., Cary, NC), and datasets were analyzed for descriptive statistics and normality using the UNIVARIATE procedure of SAS. Using the SGPLOT procedure, residuals were identified as random, homogeneous, and independent of treatment and design effects by visual inspection of distribution plots. An initial analysis of variance using the GLIMMIX procedure indicated that the fixed effects of mammary biopsy and MV side (left or right) did not affect MPF_Fick_ (*P* = 0.66) or MPF_MV_ (*P* > 0.54). As such, mammary biopsy was not included as a fixed effect in any models, MPF_MV_ was analyzed as the sum of the left and right MV, and MV diameter and TAV were analyzed as averages of the left and right MV.

The final model for MPF_Fick_, MPF_MV_, MV diameter, and TAV included the fixed effects of parity, repeated effect of day, their interaction, and the random effect of cow. A compound symmetry covariance structure was used because it yielded the lowest Akaike information criterion of the 3 covariance structures tested. A Tukey test was used to separate LSM. All values reported are LSM ± SE, and significance was declared at *P* < 0.05 and tendencies at 0.05 < *P* < 0.10. The CORR procedure was used to generate Pearson correlation coefficients between ultrasound variables and MPF_Fick_ within parity across both timepoints, as well as within parity within day. Concordance correlation coefficients (**CCC**) were calculated as a product of the Pearson correlation coefficient and a bias correction factor (Lin, 1989). The REG procedure was used to regress MPF_Fick_ on ultrasound variables (MPF_MV_, MV diameter, and TAV) by parity with day-specific intercepts. Significant models were defined as those containing linear and quadratic terms with *P* < 0.05.

Colostrum and milk production were not different between parity throughout the first 7 DIM, as described by Fischer-Tlustos et al. (2026). In brief, P2 cows produced 8.37 ± 1.01, 6.79 ± 0.53, 22.1 ± 0.81, and 20.2 ± 1.17 kg, and P3 cows produced 7.18 ± 1.30, 6.66 ± 0.63, 22.2 ± 0.65, and 21.9 ± 0.74 kg at colostrum collection, milking 2, and the morning and evening milkings at 7 DIM, respectively.

For all MPF data, there was no effect (*P* > 0.29) of a parity × day interaction; therefore, main effects will be primarily discussed herein. The effect of day of lactation on MPF_Fick_, MPF_MV_, and the associated ultrasound variables is shown in Table 1. In brief, MPF_Fick_ increased by 19.5% with advancing DIM, with MPF_Fick_ values ranging from 204 to 718 L/h and 348 to 698 L/h at calving and 7 DIM, respectively. Such increases are in agreement with previous work estimating MPF by the Fick principle in Holstein cattle from early lactation to 50 DIM (Astuti et al., 2015) and by thermodilution in goats from parturition into lactation (Reynolds, 1965; Linzell and Fleet, 1966), coinciding with associated increases in MY with advancing DIM (Kronfeld et al., 1968, Collier, 1985).

**Table 1:** Mammary plasma flow as estimated by the Fick principle (MPF_Fick_) and Doppler ultrasound of the milk vein (MPF_MV_), total milk vein blood flow (MVBF), as well as milk vein (MV) diameter and time-averaged mean velocity (TAV) as estimated via B-mode and Doppler ultrasonography, respectively, in parity-2 (n = 11) and parity-3 (n = 10) cows on the day of calving and at 7 ± 1 DIM; values represent LSM

Variable	Calving	7 DIM	SEM	*P*-value
MPF_Fick_ (L/h)	397	494	26.5	0.008
MPF_MV_ (L/h)	334	397	22.8	0.0005
Total MVBF (L/h)	478	568	32.6	0.0005
MV diameter (cm)	1.62	1.75	0.05	0.0002
MV TAV (cm/s)	31.2	32.2	1.07	0.38

As described in the methods, and in agreement with prior work (Braun and Forster, 2012), no differences were detected in ultrasound variables between the left and right MV. Thus, 3 to 5 replicates of MPF_MV_ measurement can be conducted on a single MV in a time-efficient manner (<10 min) in future work. Numerically, MPF_MV_ averages were 15.8% and 19.5% lower than MPF_Fick_ at calving and 7 DIM, respectively, in agreement with lower MPF as estimated via pudendal artery ultrasonic flow probes compared to the Fick principle (Thivierge et al., 2002) or the dye dilution technique (Bequette et al., 1999; Mabjeesh et al., 2000) in mid-to-late lactation. Similar to MPF_Fick_, MPF_MV_ increased by 15.8% with advancing DIM, with MPF_MV_ values ranging from 166 to 522 L/h and 194 to 589 L/h at calving and 7 DIM, respectively. Total MV blood flow (**MVBF**) rates are also reported at calving and 7 DIM (Table 1) to allow for comparison to previous literature. Our total MVBF value of 478 L/h at calving is highly similar to that of Braun et al. (2013; 482 L/h) at peak MVBF at 2 DIM in multiparous Swiss Braunvieh and Simmental cattle, and to that of Themistokleous et al. (2023a; 474 L/h) in multiparous Holstein cattle at peak MVBF at calving. However, no differences in MVBF with advancing DIM have previously been observed (Braun et al., 2013; Themistokleous et al., 2023a,b). Milk vein blood flow and MY are positively correlated (Braun and Forster, 2012; Themistokleous et al., 2023b), with cows producing 20 kg of milk having 41.7% greater MVBF compared to cows producing 10 kg of milk (Braun and Forster, 2012). Therefore, as milk production increased from 14.5 to 43.1 kg from the first 2 milkings after calving to 7 DIM in the present study, MPF_MV_ may increase as a consequence of mammary functional hyperemia, similar to the results observed via MPF_Fick_ measurement. However, the reasoning as to why increases in MPF_MV_ were observed from calving to 7 DIM in the present study, but not in the first week of lactation in others (Braun et al., 2013; Themistokleous et al., 2023a), requires further investigation.

Similar to MPF_MV_, MV diameter increased from 1.62 cm at calving to 1.75 cm at 7 DIM, whereas no differences were observed for MV TAV over time (Table 1). Compared to previous literature, the early-lactation MV diameters reported herein were slightly lower (2.10 cm, Braun et al., 2013; 2.39 cm, Themistokleous et al., 2023b) whereas the MV TAV values were slightly greater (26.7 to 28.2 cm/s, Braun and Forster, 2012; 27.7 cm/s, Braun et al., 2013; ∼20 to 23 cm/s, Themistokleous et al., 2023a). The present study is the first to report increases in MV diameter with advancing DIM; typically, increases in MPF_MV_ from calving into lactation are associated with increases in MV TAV (Braun et al., 2013; Themistokleous et al., 2023a). In our companion paper (Fischer-Tlustos et al., 2026), increases in MPF_MV_ from wk −4 to calving were similarly mirrored by identical increases in MV TAV, whereas MV diameter only increased at d −4 relative to calving. However, a positive correlation (r = 0.69) exists between blood flow variables and MV diameter (Braun et al., 2013). As our study is the first to detect increases in MPF_MV_ from calving to 7 DIM compared to previous literature (Braun et al., 2013; Themistokleous et al., 2023a), we speculate that as MPF_MV_ increased with increasing MY as lactation day advanced, MV diameter consequently increased to accommodate greater flow rates and pressure. Previous work has reported a lack of difference in MV diameter between cattle producing 10 or 20 kg of milk (Braun and Forster, 2012); however, such rapid increases in milk production from calving to 7 DIM in the Holstein cattle used herein may account for the simultaneous increases in MV diameter to accommodate greater rates of MPF_MV_.

No effect of parity for MPF_Fick_ was observed (*P* = 0.92; Figure 1a); however, P3 cows had greater MPF_MV_ than P2 cows (*P* = 0.01; Figure 1b). Parity-3 cows also had greater MV diameter than P2 cows throughout the sampling period (*P* = 0.008; Figure 1c), which began as early as the day of calving (*P* = 0.05; 1.76 ± 0.74 vs. 1.48 ± 0.71 cm). These findings are in agreement with Götze et al. (2010), in which German Holstein cattle entering lactation 3 or higher had greater pudendal artery area from 1 to 84 DIM and greater pudendal artery blood flow volume (as estimated via Doppler ultrasound) for the first 28 DIM compared to cattle entering lactation 2. Differences in MPF_MV_ among parity may have resulted from greater MV diameter in P3 compared to P2 cows as a result of increased vein pliability associated with greater levels of previous-lactation milk production (Kensinger et al., 1983), or greater backflow of nonmammary venous blood to the MV due to the increased risk of incompetent pudendal vein valves with advancing lactation number (Kjaesrgaard, 1974; Thivierge et al., 2000). However, because the limited replication in the present study and a lack of previous research describing the effect of parity on MV diameter and pudendal vein valve competency prevented a formal power analysis, this speculation is specific to the present group of animals and cannot be confirmed.

**Figure 1:**
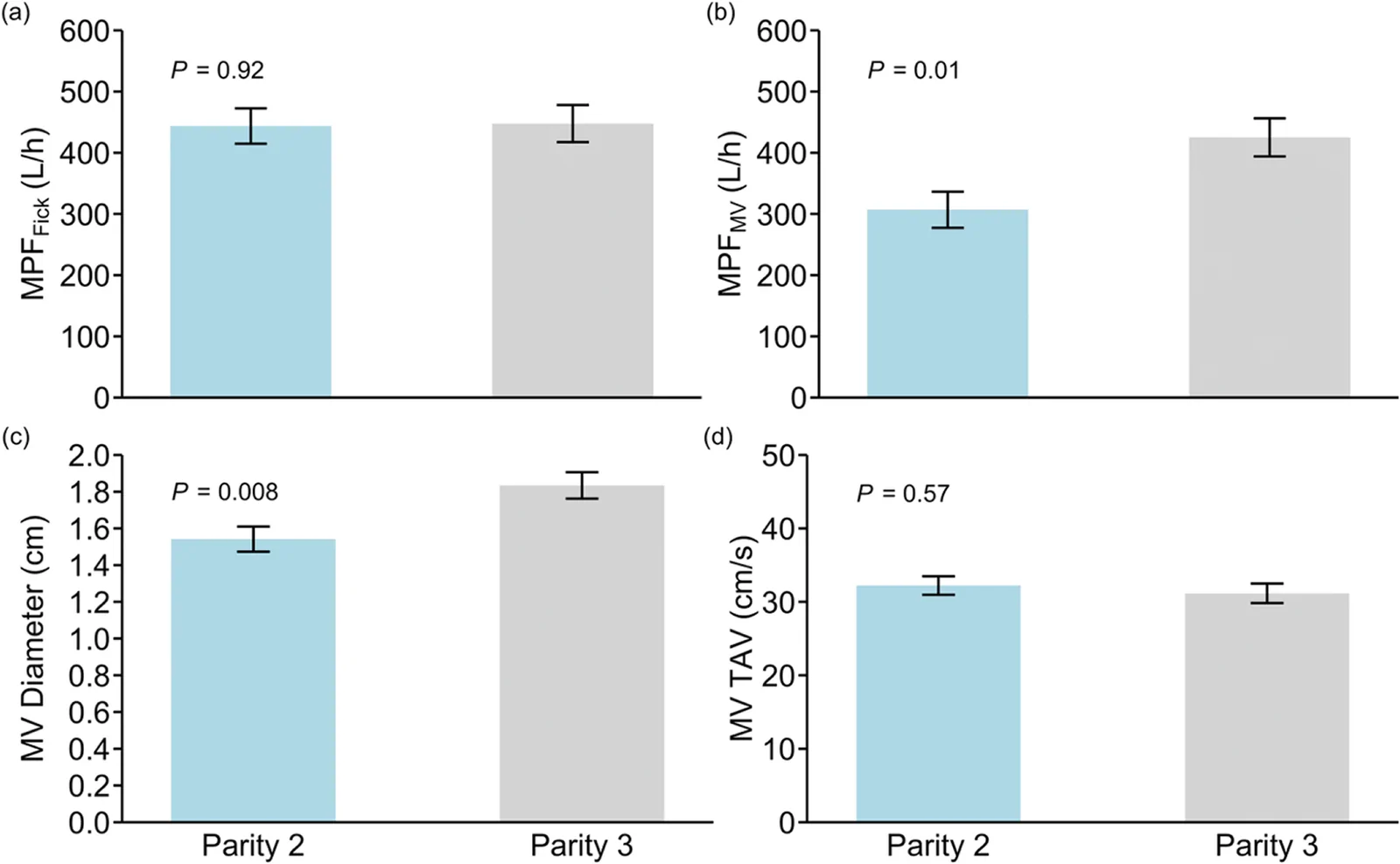
Fresh-period mammary plasma flow as estimated by (a) the Fick principle (MPF_Fick_) and (b) Doppler ultrasound of the milk vein (MPF_MV_), as well as (c) milk vein (MV) diameter and (d) time-averaged mean velocity (TAV) between parity-2 (n = 11) and parity-3 (n = 10) cows. Bars represent LSM ± SE.

Given the established correlation between MY and MPF as a consequence of functional hyperemia (Collier, 1985; Braun and Forster, 2012), such increases in MPF_MV_ with greater parity are more likely due to higher rates of previous-lactation milk production (Braun and Forster, 2012) and greater associated mammary mass of P3 compared with P2 cows, thus requiring greater rates of MPF (Kensinger et al., 1983). Although MPF_Fick_ displayed a moderate positive correlation with MY on day of calving (r = 0.60, *P* = 0.005) and at 7 DIM (r = 0.54, *P* = 0.02), MPF_MV_ exhibited a weak relationship with MY on day of calving (r = −0.26, *P* = 0.27) and at 7 DIM (r = 0.23, *P* = 0.36; Figure 2a and 2b). This is in contrast with previous work reporting a positive correlation between MY in established lactation and MVBF (Braun and Forster, 2012; Themistokleous et al., 2023b), MPF as estimated via the Fick principle (Waghorn and Baldwin, 1984; Collier, 1985; Mackle et al., 2000), and the antipyrine absorption method (Kronfeld et al., 1968). However, it is in line with Braun et al. (2013), in which a lack of relationship was detected between MVBF and MY from early lactation (calving to 10 DIM) to 300 DIM. In early lactation, a substantial portion of plasma flow is likely directed toward mammary tissue regeneration, the onset of lactogenesis II, and their associated metabolic costs (Keys et al., 1989; Capuco et al., 2001), as opposed to solely toward milk production as in established lactation, providing reasoning for the lack of a strong, positive correlation at calving and 7 DIM herein.

**Figure 2:**
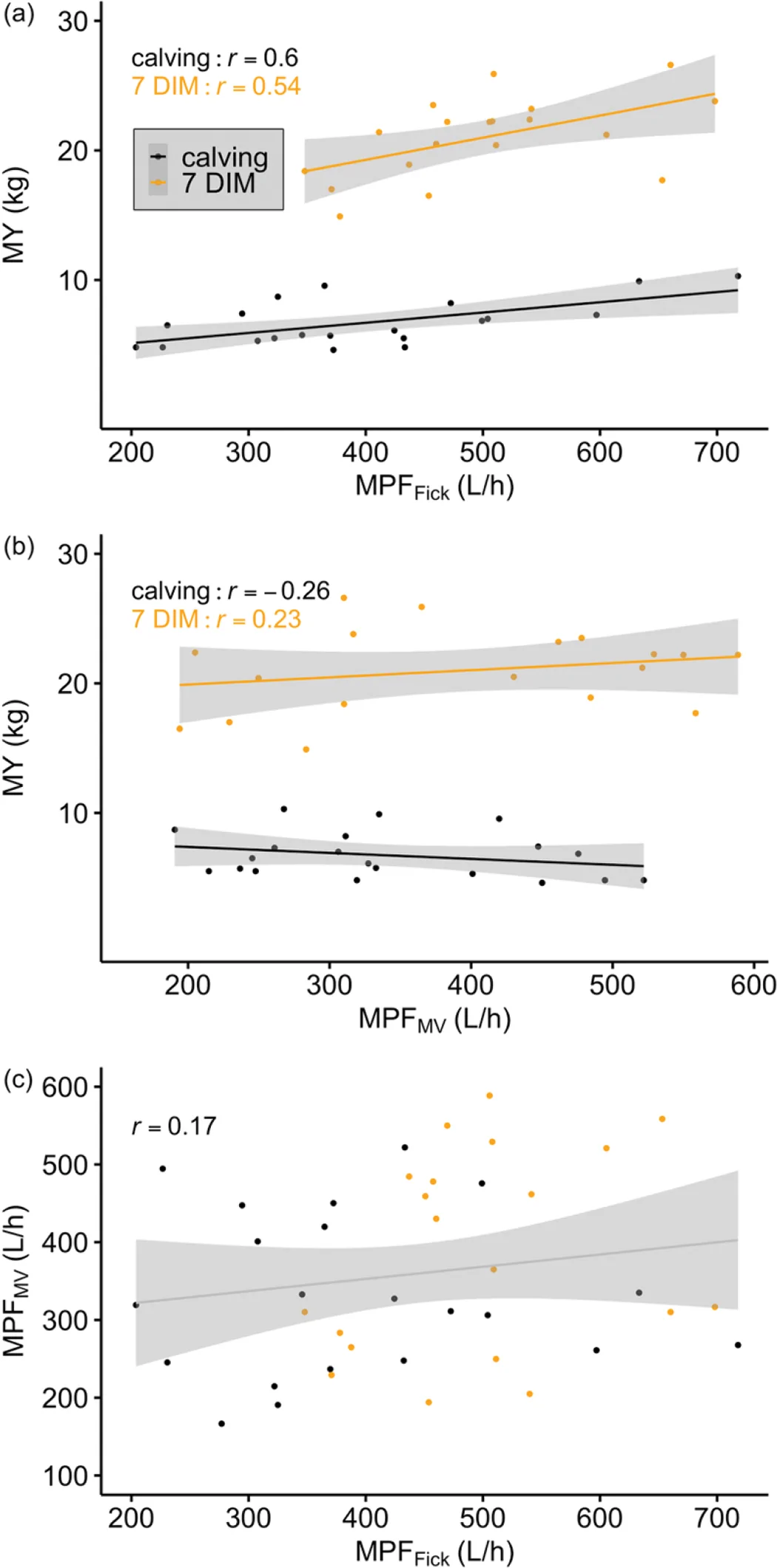
Pearson correlations (r) between (a) mammary plasma flow (MPF) estimated via the Fick principle (MPF_Fick_, L/h) and milk yield (MY, kg); (b) MPF estimated via Doppler ultrasound of the milk vein (MPF_MV_, L/h) and MY (kg); and (c) MPF_Fick_ and MPF_MV_ on the day of calving and 7 ± 1 DIM in parity-2 (n = 11) and parity-3 (n = 10) cows. The solid line represents the linear regression fit, and the shaded areas represent the 95% CI.

To the authors' knowledge, no attempts have been made to determine the relationship between MPF_MV_ and alternative measures of MPF. Doppler ultrasound variables, including MPF_MV_, MV diameter, and TAV, were not correlated with MPF_Fick_ for either P2 cows across both timepoints (r < 0.23, CCC < 0.22, *P* > 0.31) or within day of calving (r < 0.15, CCC < 0.15, *P* > 0.65) or 7 DIM (r < 0.20, CCC < 0.19, *P* > 0.55), or P3 cows across both timepoints (r < 0.12, CCC < 0.07, *P* > 0.61) or within day of calving (r < 0.27, CCC < 0.14, *P* > 0.45) or 7 DIM (r < 0.13, CCC < 0.06, *P* > 0.74; Figure 2c). In addition, no linear or quadratic (P2 cows: R^2^ < 0.14, *P* > 0.34; P3 cows: R^2^ < 0.36, *P* > 0.36) relationships were observed between MPF_Fick_ and MPF_MV_ and associated variables. A strong relationship exists between pudendal artery ultrasonic flow probes and the Fick principle in mid-to-late lactation (R^2^ = 0.96; Thivierge et al., 2002). However, whether MPF_Fick_ represents a gold standard method and the true rate of arterial MPF during the calving and fresh periods requires further discussion. The assumptions of the Fick principle state that (1) the rate of milk secretion and tissue metabolism is constant, (2) the blood flow is constant, (3) the arterial concentrations of substrates are constant, and (4) the venous sample concentration of indicator compounds is representative of the entire tissue. Sufficient mixing of mammary venous blood prior to its exit in the MV occurs due to the extreme anastomosing of mammary veins (Linzell, 1974). However, rates of milk secretion, tissue metabolism, and blood flow, as well as arterial concentrations of substrates, were likely not constant during the 12 h of MPF_Fick_ measurement. Specifically, the calving and fresh periods are characterized by a myriad of hormonal, immunological, and metabolic changes that may have resulted in the violation of 3 out of 4 assumptions of the Fick principle. Such violations of the Fick principle assumptions may provide reasoning as to why parity differences could not be detected with MPF_Fick_ but were detected with MPF_MV_. In contrast to the Fick principle, the assumptions proposed for accurate MPF_MV_ measurement were that (1) an acclimated, stress-free state has been achieved prior to and is maintained during measurement; (2) blood flow is constant; and, (3) MV diameter remains constant during measurement. Although researchers took precaution to ensure cows were acclimated to the procedure, this technique may have been subject to changes in physiological rather than behavioral stress, which likely occur with the onset of high milk production and its associated adaptations, as described for MPF_Fick_. Regarding assumptions 2 and 3, potential intracow variation in MPF_MV_ and MV diameter was likely reduced by conducting 3-5 replicates per MV per sampling time. However, intercow variation in the proportion of mammary venous blood flowing through the MV compared to the pudendal vein may have occurred. Mammary venous drainage in cattle occurs primarily through the MV (Braun and Forster, 2012); yet, the manual clamping of the pudendal vein can alter MVBF by more than 10% in 37.5% of older, late-lactation cows (Kjœrsgaard, 1974). Thus further investigation is required into whether the proportion of venous blood flowing through the MV is constant between cows, and the potential risk factors for cattle that may have altered or inconsistent proportions of mammary venous blood exiting via the MV compared to the pudendal vein.

In conclusion, the expected trends were observed in MPF_MV_ and MPF_Fick_ from calving to 7 DIM, demonstrating that assumptions of each technique were not violated to such an extent that the expected changes in MPF could not be observed. However, no relationship was detected between the MPF_MV_ and MPF_Fick_ techniques at calving and 7 DIM; thus, validation of MPF_MV_ using the common technique of MPF_Fick_ could not be conducted herein. Importantly, our MPF_MV_ results were in agreement with previous research, demonstrating that 3 to 5 replicates of MPF_MV_ measurement can be conducted in a time-efficient, noninvasive manner on a single MV. Future work should aim to assess MPF_MV_ measurement with techniques that assess MPF directly in the pudendal artery, such as the dye dilution or ultrasonic flow probe techniques. Validation of Doppler ultrasound of the MV to estimate MPF could result in widespread implementation, allowing for greater insight into strategies that influence fresh-period mammary metabolism and milk production directly at the gland level.
